# Green Tea Extract Ameliorates Learning and Memory Deficits in Ischemic Rats via Its Active Component Polyphenol Epigallocatechin-3-gallate by Modulation of Oxidative Stress and Neuroinflammation

**DOI:** 10.1155/2012/163106

**Published:** 2012-08-02

**Authors:** Kuo-Jen Wu, Ming-Tsuen Hsieh, Chi-Rei Wu, W. Gibson Wood, Yuh-Fung Chen

**Affiliations:** ^1^School of Chinese Pharmaceutical Sciences and Chinese Medicine Resources, College of Pharmacy, China Medical University, 91 Hsueh-Shih Road, Taichung 40402, Taiwan; ^2^Department of Pharmacology, University of Minnesota and Geriatric Research, Education and Clinical Center, VA Medical Center, Minneapolis, MN 55455, USA; ^3^Department of Pharmacology, College of Medicine, China Medical University, 91 Hsueh-Shih Road, Taichung 40402, Taiwan; ^4^Department of Pharmacy, China Medical University Hospital, Taichung 40421, Taiwan

## Abstract

Ischemic stroke results in brain damage and behavioral deficits including memory impairment. Protective effects of green tea extract (GTex) and its major functional polyphenol (−)-epigallocatechin gallate (EGCG) on memory were examined in cerebral ischemic rats. GTex and EGCG were administered 1 hr before middle cerebral artery ligation in rats. GTex, EGCG, and pentoxifylline (PTX) significantly improved ishemic-induced memory impairment in a Morris water maze test. Malondialdehyde (MDA) levels, glutathione (GSH), and superoxide dismutase (SOD) activity in the cerebral cortex and hippocampus were increased by long-term treatment with GTex and EGCG. Both compounds were also associated with reduced cerebral infraction breakdown of MDA and GSH in the hippocampus. In *in vitro* experiments, EGCG had anti-inflammatory effects in BV-2 microglia cells. EGCG inhibited lipopolysaccharide- (LPS-) induced nitric oxide production and reduced cyclooxygenase-2 and inducible nitric oxide synthase expression in BV-2 cells. GTex and its active polyphenol EGCG improved learning and memory deficits in a cerebral ischemia animal model and such protection may be due to the reduction of oxidative stress and neuroinflammation.

## 1. Introduction

Ischemic stroke results from a temporary or permanent reduction of cerebral blood flow that leads to functional and structural damage in different brain regions. Cellular damage occurs during ischemia [1, 2] and reperfusion [3, 4]. Deleterious effects include ATP depletion, intracellular calcium changes, loss of ion homeostasis, excitotoxicity, activation of enzymes, arachidonic acid release, and mitochondrial dysfunction [5, 6].

These changes are associated with increased production of reactive oxygen species (ROS) which can cause severe oxidative damage to brain tissue [7]. Superoxide dismutase (SOD), glutathione peroxidase (GSH-Px), and catalase (CAT) are involved in the intracellular defense against ROS [8]. ROS are usually scavenged by antioxidant enzymes, such as SOD. SODs catalyze the production of O_2_ and H_2_O_2_ from superoxide (O_2_^−^) followed by catalase and glutathione-peroxidase-catalyzed decomposition of hydrogen peroxide into water [9]. Subsequently, reperfusion can trigger inflammation mediated by phospholipases, COX-2, and nitric oxide synthases (NOSs) [5, 6].

Some brain regions, such as the striatum and hippocampus, are more vulnerable to ischemic damage [10]. CA1 hippocampal pyramidal neurons exhibit cell death several days after ischemic injury [11]. Spatial memory in rats and humans is largely dependent on the hippocampus [12] and hippocampal neuronal damage induced by ischemia is associated with spatial memory impairment. Microglia is widely distributed throughout large nonoverlapping regions of the central nervous system [13, 14]. Microglia is sensitive to even small pathological changes and is traveling within the brain [15, 16] and will be stimulated to proliferate when the brain or tissues are damaged. They are constantly cleaning damaging neurons, plaques, and infectious pathogens, to stop potentially fatal injuries [17]. Over the past decade, they are considered as a modulator of neurotransmission, although the mechanisms are not yet fully understood [18, 19]. Murine BV-2 microglia cells were consciously used to study the bioactivities of neuroprotection, synthases, and cytokine of microglia cells [20–22].

Green tea was neuroprotective in ischemia-reperfusion brain injury in rats and gerbils [23–25]. The main catechins in green tea are (−)-epicatechin; (−)-epicatechin gallate (ECG); (−)-epigallocatechin (EGC); (−)-epigallocatechin gallate (EGCG). EGCG is the most active polyphenol in green tea [26]. EGCG has antioxidative [27], anticancer [28], and anti-inflammatory effects [29, 30]. Many studies have reported that EGCG had neuroprotective effects in animal models of cerebral ischemia [31–34] which may be attributed to its antioxidant and free radical scavenging actions. There have been few studies reporting on the effects of green tea and its main component, EGCG on memory in an animal model of cerebral ischemia. Therefore, we determined if green tea extract and EGCG would reduce memory impairment in a rat model of cerebral ischemia. Effects of green tea extract and EGCG on neuroinflammation in LPS-induced BV-2 microglia cells were also examined.

## 2. Materials and Methods

### 2.1. Preparation of Green Tea Extracts

Green tea (*Camellia sinensis (*L.*) O. Kuntze*) was provided by Mr. Tsung-Chih Wu of the Kuo-Ming Tea Factory, Nantou, Taiwan. Fresh tea leaves (3000 g) were immersed in 10 L distilled water and were extracted using 85°C water for 12 hr and repeated twice. The extracts were filtered and freeze-dried. The yield percentage of green tea extract (GTex) was 217 g and 7.23% of the total.

### 2.2. Reagents and Chemicals

(−)-Epicatechin, (−)-epicatechin gallate (ECG), (−)-epigallocatechin (EGC), (−)-epigallocatechin gallate (EGCG), caffeine,* tert*-butylhydroquinone(BHQ), acetic acid, pentoxifylline (PTX), N-methyl-2-phenylindole (NMPI), tetramethoxy propane (TMP), lipopolysaccharide (LPS), and Griess reagent were purchased from Sigma-Aldrich (St. Louis, MO, USA). Zoletil was purchased from Virbac Laboratories (Carros, France). BCA Protein assay kit was purchased from Thermo Fisher Scientific (Lafayette, CO, USA). MDA-586 assay Kit and Glutathione (GSH) assay kit were purchased from Cayman Chemical (Ann Arbor, MI, USA). Anti-iNOS antibody (rabbit polyclonal to iNOS, sc-651) and anti-COX-2 antibody (rabbit polyclonal to COX-2, sc-7951) were purchased from Santa Cruz Biotechnology (Santa Cruz, CA, USA).

### 2.3. Determination of Polyphenol Compounds by HPLC

The quantification of polyphenol compounds was using a HPLC procedure following the previous report [35]. GTex was dissolved in methanol and then filtered with a 0.22 *μ*m membrane filter (Millipore, MA, USA). Stock solutions of the standards were prepared in methanol to final concentrations of 1 mg/mL. All standard and sample solutions were injected into 20 *μ*L in triplicate. The Shimadzu VP series HPLC system and Shimadzu Class-VP chromatography data system were used. All chromatographic operations were carried out at 25°C. The chromatographic peaks of polyphenol compounds were confirmed by comparing their retention times and UV spectra. A LiChrospher RP-18e (250 × 4 mm, 5 *μ*m) column (Merck KGaA, Darmstadt, Germany) was used. Chromatographic separations of polyphenol compounds, including (−)-epicatechin, (−)-ECG, (−)-EGC, (−)-EGCG, and caffeine, were carried out using a two-solvent system: solvent A 100% methanol and solvent B 0.2% acetic acid at pH = 3.23. The analyses were performed using a gradient program. The conditions were as follows: initial condition of 90% solvent B, 0–5 min changed to 80% solvent B, 5–30 min unchanged, 30–50 min changed to 50% solvent B, 50–55 min changed to 40% solvent B, and 55–60 min unchanged. Signals were detected at 280 nm. *tert*-butylhydroquinone (BHQ, 25 *μ*g/mL) was used as an internal standard. Quantification was carried out using standard calibration curves. The concentrations used for the calibration of reference polyphenol compounds were between 10 and 150 *μ*g/mL.

### 2.4. Animals and Drug Administration

Male Sprague–Dawley (SD) rats, 8-9 wks of age, weighing 250–300 g, were purchased from BioLASCO Taiwan Co., Ltd. Rats were fed normal rat chow and housed in standard cages at a constant room temperature of 22 ± 1°C, with humidity 55 ± 5% and a 12 hr inverted light-dark cycle for at least 1 week before the experiment. The experimental protocol was approved by the Institutional Animal Care and Use Committee (IACUC), China Medical University, protocol 100–220-C. The minimum number of animals and duration of observations required to obtain reliable data were used. For infarct size evaluation studies, the animals were divided into seven groups of six animals each: the ischemia/reperfusion induction group (I/R; as a control group), treatment with GTex (30, 100, and 300 mg/kg) groups, and EGCG (10 mg/kg) group. GTex and EGCG were dissolved in distilled water and administered orally 1 hr before cerebral artery ligation.

**Figure 1:**
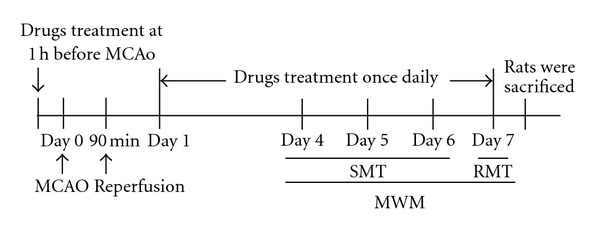
Schedule of drug treatment and experiment orders. Green tea extract (GTex), EGCG, and PTX were administrated orally 1 h before the surgery. The oral administration to rat continued once daily for 7 days and 1 h prior to training or testing. Four to seven days after surgery, the spatial memory test (SMT) of the Morris water maze (MWM) was performed 4 trials a day for 3 consecutive days, followed 24 h later (day 7) by the reference memory test (RMT). Rats were sacrificed immediately after the behavioral test.

For behavioral studies, the animals were divided into seven groups of six animals each: the sham operation group (sham; as a normal group), the ischemia/reperfusion induction group (I/R; as a control group), treatment with GTex (30, 100, and 300 mg/kg) groups, EGCG (10 mg/kg) group, and PTX (100 mg/kg) group. Drugs were dissolved in distilled water and administered orally 1 hr before ischemia occlusion and once daily during the duration of the experiment. Four days after ischemia/reperfusion surgery, the rats were given behavioral training in a Morris water maze. The schedule for drug treatment, surgery, and behavioral testing is shown in Figure 1.

### 2.5. Transient Focal Cerebral Ischemia-Reperfusion Model

Focal ischemia was induced by occlusion of the right middle cerebral artery (MCA) and both common carotid arteries (CCAs) as previously described [36]. Briefly, all rats were fasted overnight with free access to water and then anesthetized with zoletil (25 mg/kg, i.p.) and the skull exposed and a small burr hole was made over the MCA. A 10–0 nylon monofilament (Davis & Geck, Wayne, NJ, USA) was placed underneath the right MCA rostral to the rhinal fissure, proximal to the major bifurcation of the right MCA, and distal to the lenticulostriate arteries. The artery then was lifted, and the wire rotated clockwise. Both CCAs were then occluded using a microvascular clip (FE691; Aesculap, Tuttlingen, Germany). Reperfusion was established after 90 minutes of occlusion by first removing the microvascular clips from the CCA, then rotating the wire counterclockwise, and removing it from beneath the MCA.

### 2.6. Infarct Volume Measurement

The rats were deeply anesthetized by intraperitoneal dose of 50 mg/kg of zoletil; intracardiac perfusion with 200 mL of freezing PBS was performed before animals were decapitated. The brain was removed and sliced in 2 mm sections using a rodent brain matrix slicer (RBM-4000C; ASI Instruments, Warren, MI, USA). The sections were stained with 2% 2,3,5-triphenyltetrazolium chloride (TTC) for 10 min at room temperature and fixed in 10% formalin. The image of each section was digitized and the infarct volumes were determined morphometrically using Image-Pro Plus 6.0 (Media Cybernetics, MD, USA).

### 2.7. Morris Water Maze Test

Behavioral testing was performed in water maze. The apparatus consisted of a round water tank with a transparent platform stand inside. The transparent platform was submerged 1 cm below the water level and located in a constant position in the middle of one quadrant, equidistant from the center and edge of the pool. For each training session, the rats were put into the water at one of four starting positions, the sequence of the positions being selected randomly. In each training session, the latency to escape onto the hidden platform was recorded with a camera fixed on the ceiling of the room and images stored in a computer. In the hidden-platform test, the rats were given four trials per day [37, 38]. Training was conducted for 3 consecutive days (Morris Water Maze spatial memory test on treatment day 4–6). During each trial, the rats were released from four pseudorandomly assigned starting points and allowed to swim for 120 s. After mounting the platform, the rat was allowed to remain on the platform for 30 s. The rat was then placed in the home cage until the start of the next trial. The rat would be guided to the platform and would be allowed to rest on the platform for 30 s, if the rat was unable to find the platform within 120 s. In the probe trial, the hidden platform was removed, and the animal was allowed to float freely for 60 s. The parameters measured during the probe trial were the time spent in the quadrant of the target platform (Morris Water Maze reference memory study on treatment day 7).

### 2.8. Biochemical Assays

#### 2.8.1. Biochemical Examinations

At the end of the behavioral test, rats were sacrificed using zoletil (50 mg/kg, i.p.) for biochemical studies. Brains were quickly removed and the cerebral cortex and hippocampus were separated on ice. To prepare a homogenate, brain tissue was mixed with 0.1 M phosphate buffer saline (PBS, pH = 7.4) and centrifuged at a 10,000(g) at 4°C for 15 min to remove cellular debris. The supernatant was used for the estimation of the following malonyldialdehyde (MDA) levels, SOD activity, and GSH levels. Protein concentration of samples was determined by BCA Protein assay kit with BSA used as a standard.

#### 2.8.2. Measurement of Malonyldialdehyde (MDA) Level

Malonyldialdehyde (MDA) was determined spectrophotometrically using the *N*-methyl-2-phenylindole (NMPI) method of Bergman [39]. Fifty *μ*L sample or standard was added and followed by 160 *μ*L of 10 mM solution of NMPI. A similar approach was used for the standard; TTMP (tetramethoxy propane) was used at concentrations from 0.8 to 8 *μ*M. The plate was incubated for 48 min at 45°C. The chromophore absorbs at 586 nm.

#### 2.8.3. Measurement of Superoxide Dismutase (SOD) Activity

Superoxide dismutase (SOD) activity was based on the inhibitory effect of SOD on the reduction of nitroblue tetrazolium (NBT) by the superoxide anion generated by the system xanthine/xanthine oxidase, measuring the absorption at 560 nm [40].

#### 2.8.4. Measurement of Glutathione (GSH) Level

Glutathione (GSH) levels were determined spectrophotometrically using the DTNB-GSH reductase recycling method, measuring the absorption at 405 nm [41]. 

### 2.9. Cell Culture

#### 2.9.1. BV-2 Cell Culture

The murine microglial BV-2 cell line was provided by Professor Jau-Shyong Hong from the Neuropharmacology Section Lab of Pharmacology and Chemistry, NIEHS/NIH, Bethesda, USA. The BV-2 cells were maintained in DMEM supplemented with 10% FBS. One hundred U/mL of penicillin and 100 *μ*g/mL streptomycin were added to DMEM, and the cells were kept at 37°C in a humidified incubator under 5% CO_2_ and 95% air.

#### 2.9.2. Nitrite Assay

Nitrite, the stable metabolite of NO, was assayed as the production of NO in the culture medium, and the accumulation of nitrite in the medium was determined by colorimetric assay with Griess reagent. 1 × 10^4^ BV-2 cells were seeded in each well of 96-well plates and kept overnight. Cells were then changed to phenol-red free DMEM. The BV-2 cells were pretreated with EGCG for 1 hr and then stimulated with 0.5 *μ*g/mL LPS. After further 24 h of incubation, 100 *μ*L of culture supernatant reacted with an equal amount of Griess reagent (1% sulfanilamide in 5% H_3_PO_4_ and 0.1% N-1-naphthylethylenediamide dihydrochloride) in 96-well culture plates for 10 min at room temperature in the dark. Nitrite concentrations were determined by using standard solutions of sodium nitrite prepared in cell-culture medium. The absorbance at 550 nm was determined using an ELISA reader [42]. Each experiment was performed in triplicate.

#### 2.9.3. Preparation of Cell Extracts

The test medium was removed from culture dishes, and cells were washed with ice-cold PBS. The cells were scraped, resuspended in lysis buffer, then centrifuged at 12,000 (rpm) for 30 min at 4°C. Protein concentrations of samples were determined by the BCA Protein assay kit with BSA as a standard.

#### 2.9.4. Western Blotting

Samples containing 70 *μ*g of protein were separated on 10% SDS-PAGE (sodium dodecyl sulfate-polyacrylamide gel electrophoresis) and transferred to PVDF (polyvinylidene difluoride) membranes. The membranes were incubated for 1 hr with 5% dry skim milk in TBST buffer at room temperature to prevent nonspecific binding. The membranes were then incubated with rabbit anti-iNOS (1 : 1000) and rabbit anti-COX-2 (1 : 1000). Subsequently, the membranes were incubated with goat anti-rabbit alkaline-phosphatase-conjugated secondary antibody (1 : 1000) for 1 hr at room temperature. Bands were visualized using the chromogenic substrate 5-bromo-4-chloro-3-indolyl phosphate in the presence of nitroblue tetrazolium. 

### 2.10. Statistical Analysis

All data were expressed as the mean ± standard error. Data were analyzed using either Student's *t*-test or one-way ANOVA followed by Dunnett's test. *P* < 0.05 was considered significant.

## 3. Results

### 3.1. Composition and Stability of Polyphenol Compounds in GTex

**Figure 2:**
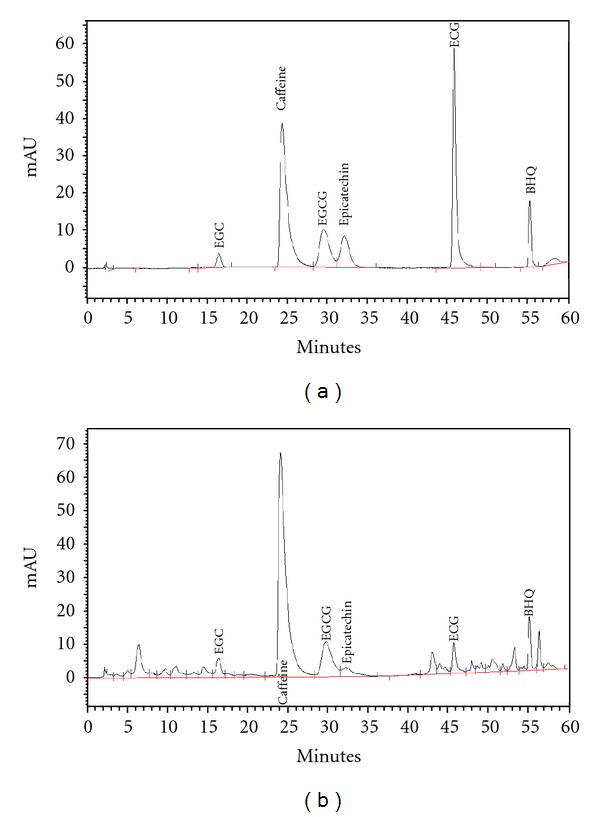
HPLC chromatograms of the GTex at 280 nm. Trace: (a) standard, (b) GTex. BHQ: *tert*-butylhydroquinone as an internal standard. (EGCG: (−)-epigallocatechin gallate, ECG: (−)-epigallocatechin, EGC: (−)-epicatechin gallate).

Analysis of GTex by HPLC indicated that the total green tea solids in the extract contained (−)-epigallocatechin gallate (3.21%), (−)-epigallocatechin (4.59%), (−)-epicatechin gallate (1.06%), (−)-epicatechin (1.31%), and caffeine (4.46%) as shown in Figure 2 and Table 1.

### 3.2. Effects of GTex and EGCG on Cerebral Infarct Volume

**Figure 3:**
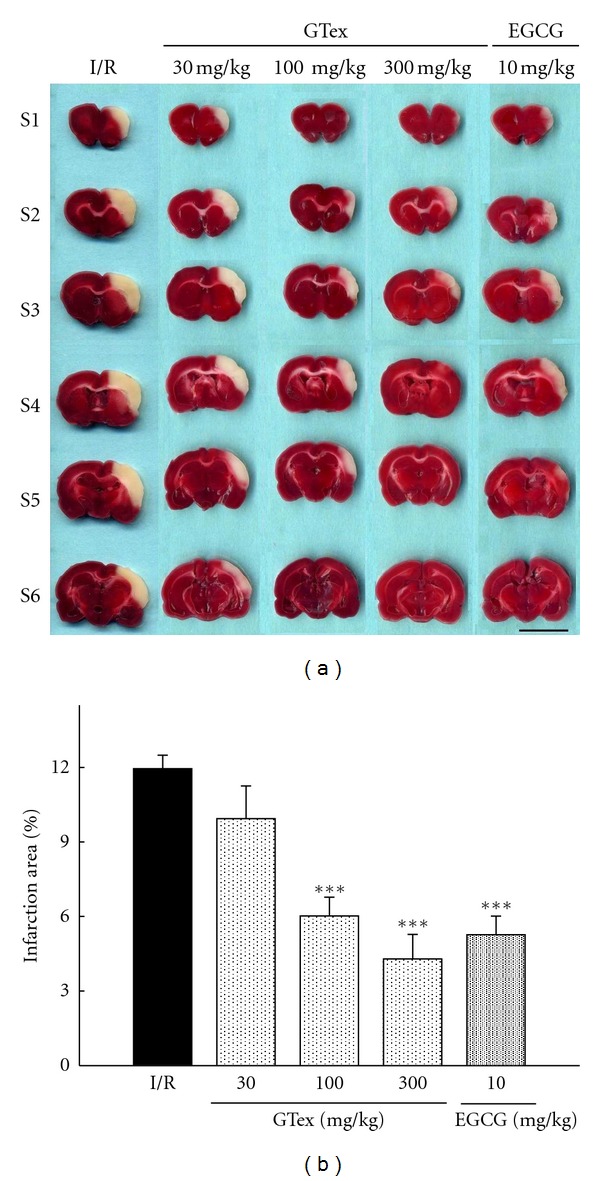
Effects of GTex and EGCG on cerebral infarction. (a) Effect of GTex (30~300 mg/kg, p.o.) groups and EGCG (10 mg/kg, p.o.) on cerebral infarct area at 24 h after reperfusion. The pale area represents infarct tissue and the red area normal tissue. (b) Infarction area by TTC staining (*n* = 6 in each group). I/R: ischemia/reperfusion control group. Each vertical bar represented mean ± S.E. *P < 0.05, ***P < 0.001 compared to I/R group. Scale bar = 1 cm.

It can be seen in Figure 3 that visible boundaries were clearly observable between normal brain tissue and untreated cerebral infarct tissue. GTex treatment (100 and 300 mg/kg) markedly reduced cerebral infarction at 24 hr after reperfusion as compared with the ischemia/reperfusion (I/R) group (Figure 3(a)). The percent infarct size was 11.9 ± 0.54% in the untreated group and 6.0 ± 0.76% and 4.3 ± 0.99% in the 100 and 300 mg/kg GTex treatments, respectively (Figure 3(b)). EGCG also significantly reduced infarct size (*P* < 0.001) (Figure 3(b)) as compared with the I/R group but no EGCG. The sizes of the cerebral infarction in the GTex and EGCG groups were similar.

### 3.3. Effects of GTex, EGCG, and PTX on Spatial Performance Memory in Ischemic Rats

**Figure 4:**
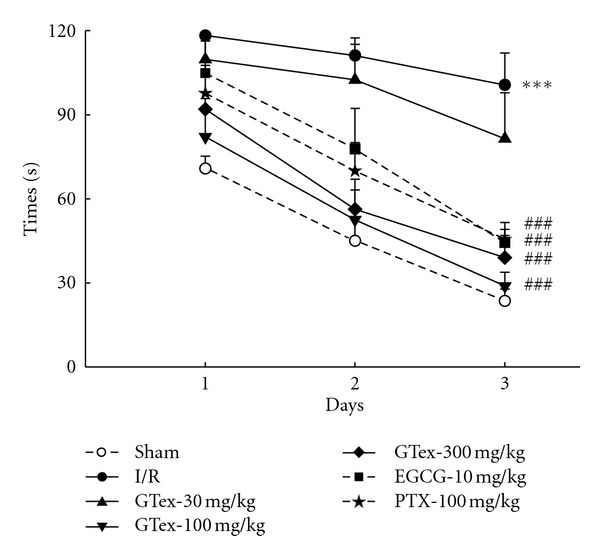
Effect of GTex (30~300 mg/kg, p.o.), EGCG (10 mg/kg, p.o.), and pentoxifylline (PTX, 100 mg/kg, p.o.), on the swimming time took to reach the hidden platform of the Morris water maze in the ischemia/reperfusion (I/R) rats. **P < 0.01, ***P < 0.001 compared to the sham group. ^#^ *P* < 0.05, ^##^ *P* < 0.01, ^###^ *P* < 0.001 compared to I/R group (*n* = 6 in each group).

The sham group quickly learned the location of the platform as demonstrated by a reduction in escape latencies on days 1 and 2 and by reaching stable latencies on day 3 (Figure 4). Furthermore, we found the swimming pathway required to reach the submerged platform was simplified in the sham group. By contrast, in the I/R group, a typical swimming behavior consisted of circling around the pool and the escape latencies in trials 1 and 2 remained essentially unchanged throughout the 3-day testing period. GTex (100 and 300 mg/kg) treatment significantly improved performance (i.e., reduced escape latency) of ischemic/reperfusion rats on the escape latency on day 2 (*P* < 0.01) and day 3 (*P* < 0.001) testing periods. EGCG (10 mg/kg) and PTX (100 mg/kg) treatment reduced the escape latency in the day 2 (*P* < 0.05) and day 3 (*P* < 0.001) testing periods.

### 3.4. Effects of GTex, EGCG, and PTX on Time in the Target Quadrant

**Figure 5:**
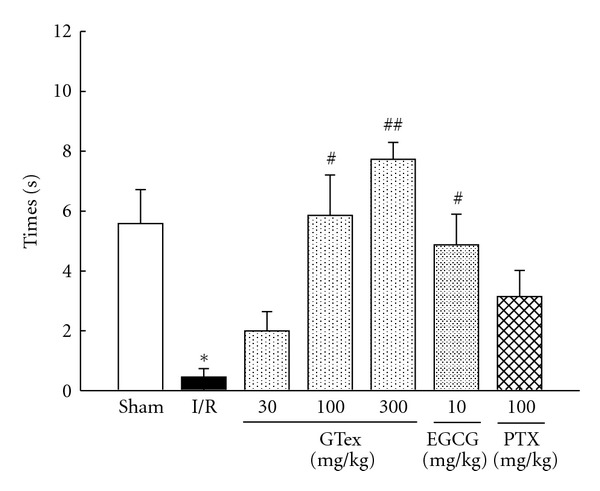
Effect of GTex (30~300 mg/kg, p.o.), EGCG (10 mg/kg, p.o.), and pentoxifylline (PTX, 100 mg/kg, p.o.), on the time spent in the target quadrant in ischemia/reperfusion (I/R) rats. The performance of each rat was tested 24 hours after the final training day in a probe trial (60 sec) during which the platform was removed. *P < 0.05 compared to the sham group. ^#^ *P* < 0.05, ^##^ *P* < 0.01 compared to I/R group (*n* = 6 in each group).

It can be seen in Figure 5 that the time in the target quadrant in the I/R group was significantly reduced compared to that of the sham group (*P* < 0.05). GTex (100 and 300 mg/kg) significantly reduced ischemia/reperfusion- induced time in the target quadrant when administered before the training trial (*P* < 0.05–0.01) as compared with the I/R group. EGCG (10 mg/kg) had similar effects as GTex but PTX did not improve performance (Figure 5).

### 3.5. MDA Levels in Cortex and Hippocampus

MDA levels in the cortex and hippocampus of the different groups are shown in Table 2. MDA levels were significantly increased in the I/R group (*P* < 0.001) as compared with the sham group. In contrast, MDA levels were decreased significantly after treatment with GTex (300 mg/kg) (*P* < 0.001) and EGCG (10 mg/kg) (*P* < 0.05–0.001, Table 1). GTex at lower dosage (30 and 100 mg/kg) and PTX (100 mg/kg) did not alter MDA levels in the cortex and hippocampus of the rats as compared with the I/R group with an exception that GTex 100 mg/kg significantly reduced MDA levels in the hippocampus (*P* < 0.01). 

### 3.6. SOD Activity in Cortex and Hippocampus

There were no significant differences in SOD activity in brain tissue of I/R and sham animals. However, SOD activity was significantly decreased after treatment with GTex (300 mg/kg) and EGCG (10 mg/kg) in the cortex (*P* < 0.05) and hippocampus (*P* < 0.01) when compared with the I/R group. The lower GTex concentrations (30 and 100 mg/kg) and PTX (100 mg/kg) did not significantly change SOD activity as compared with the I/R group (Table 3).

### 3.7. GSH Levels in Cortex and Hippocampus

GSH levels were significantly decreased in the cortex and hippocampus (Table 4) of the I/R group (*P *<0.001). After treatment with GTex (100 and 300 mg/kg) and EGCG (10 mg/kg), GSH levels were significantly increased in the cortex (*P* < 0.01) and hippocampus (*P *< 0.001). GTex at the lowest concentration tested (30 mg/kg) and PTX (100 mg/kg) did not significantly change GSH levels in the rat cortex and hippocampus.

### 3.8. Effects of EGCG on LPS-Induced NO Production in BV-2 Cells

**Figure 6:**
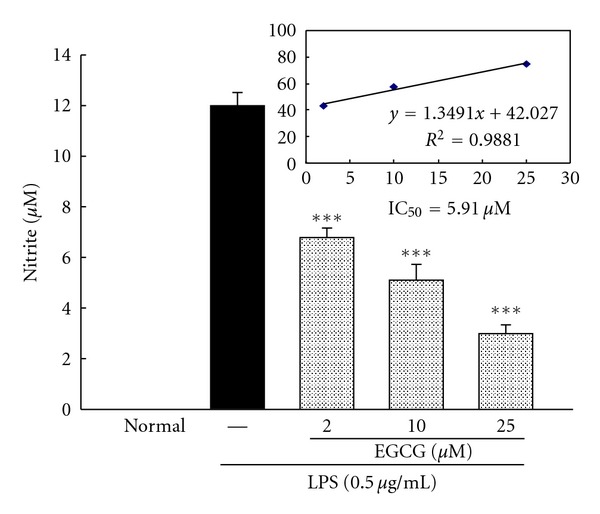
Inhibitory effect of EGCG on LPS-induced NO production in BV-2 cells incubated with LPS (0.5 *μ*g/mL) in the presence or absence of indicated concentration of EGCG. Accumulated nitrite in the culture medium was determined by the Griess reaction. Each vertical bars represented mean ± S.E. ***P < 0.001 compared to LPS only group.

BV-2 cells incubated with 0.5 *μ*g/mL LPS displayed a significant increase in nitrite production as compared with sham controls (Figure 6). EGCG in a concentration-dependent manner significantly reduced LPS-induced nitrite production (Figure 6). The IC_50_ for ECGC on inhibition of LPS-induced nitrite production was 5.91 *μ*M in BV-2 cells (Figure 6).

### 3.9. Effects of EGCG on Expression of COX-2 and iNOS in BV-2 Cells

**Figure 7:**
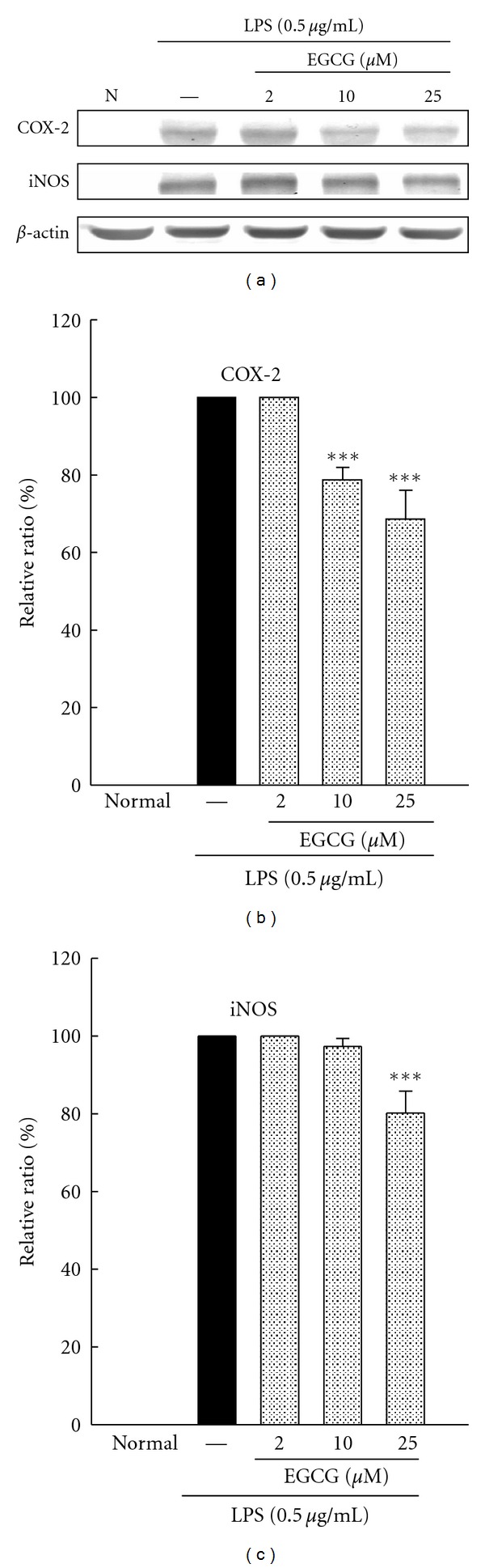
Effects of EGCG (2, 10, and 25 *μ*M) on expression of COX-2 and iNOS in BV-2 cells treated with lipopolysaccharide (LPS, 0.5 *μ*g/mL) for 24 h. Cultures were pretreated with EGCG for 1 h before the addition of LPS treatment. Bars represent the mean ± SE from three independent experiments. Densitometry analyses are presented as the relative ratio of protein/*β*-actin protein and are represented as percentages of the LPS only group. ***P < 0.001 compared to LPS only.

Changes in protein abundance of COX-2 and iNOS induced by LPS were measured at by Western blot analysis. Elevated COX-2 and iNOS protein production were detected at 24 hr following LPS treatment. LPS-induced iNOS and COX-2 expression were significantly suppressed by EGCG pretreatment at concentrations of 10 and 25 *μ*M but not at a lower concentration of 2 *μ*M (Figure 7). 

## 4. Discussion

Cerebral ischemia causes cognitive deficits, including memory impairment [43, 44]. The Morris water maze is a widely used test in behavioral neuroscience for studying the neural mechanisms of spatial learning and memory. Cerebral ischemia has been reported to produce deficits in memory performance in the Morris water maze [37]. Our results showed that cerebral ischemia induced impairment in both spatial memory and reference memory in a Morris water maze and is in agreement with previous studies [43, 44]. GTex (100 and 300 mg/kg) markedly improved deficits in spatial memory induced by cerebral ischemia. In addition, cerebral ischemia-induced reference memory deficits were also blocked by treatment with GTex. We also found that oral administration of EGCG for 7 days could reduce deficits in spatial and reference memory in rats of the ischemic group. EGCG is a major component of GTex and our results suggest that improved memory observed in GTex rats may be attributable to EGCG, although other GTex active compounds cannot be ruled out. There are reports that EGCG improved learning and memory in animal models of Alzheimer's disease and diabetes [45, 46]. EGCG did not reduce deficits in learning and memory deficits induced by cerebral ischemia in another study report [47]. There are several differences between the present study and the earlier report. In the earlier study, a 4-VO (four-vessel occlusion) model was used to restrict the cerebral circulation for ten minutes, two times within 60 min. Also, 50 mg/kg of EGCG was given intraperitoneally 30 min before the first occlusion. We used a 3-VO (three-vessel occlusion) model to induce ischemia/reperfusion damage and 10 mg/kg of EGCG was orally administered once daily for 7 days. In the current study, repeated administration of EGCG (10 mg/kg) improved both spatial memory and reference memory in a water-maze test. Results from the present experiments indicated that EGCG improved learning and memory in an animal model of ischemia rodents and required long-term treatment.

The present study evaluated the neuroprotective effects of GTex and EGCG in an ischemic stroke animal model and the anti-inflammatory effects of EGCG in BV-2 cells. GTex administered *in vivo* was effective in reducing damage in a stroke model. Treatment with GTex (100 and 300 mg/kg) significantly reduced cerebral infraction at 90 min ischemic occlusion and 24 hr reperfusion. The present studies showed that green tea had a neuroprotective effect in a transient focal ischemia model in agreement with previous studies [23–25]. EGCG also had similar effects and those results are consistent with previous reports [31, 48]. 

It has been reported that oxygen free radical-induced lipid peroxidation plays an important role in the neurological damage occurring after cerebral ischemia [49]. We found that 7 days following cerebral ischemia MDA levels were significantly increased as compared with levels in sham-operated rats. Administration of GTex and EGCG reversed the spike in MDA levels seen in the cerebral ischemic rats. GTex and EGCG may act by scavenging oxygen free radicals. Reactive oxygen species (ROS) are produced continuously *in vivo* under aerobic conditions. GSH-Px, CAT, and SOD, along with GSH and other nonenzymatic antioxidants act in concert to protect brain cells against oxidative damage. ROS are contributors to ischemic brain damage [49]. SOD is involved in the regulation of antioxidant defenses by catalyzing the dismutation of superoxide anion into H_2_O_2_ and O_2_. Candelario-Jalil et al. [50] showed that SOD activity was increased at 24~72 hr after cerebral ischemia then returned to normal after 96 hr. We found that SOD activity 7 days after cerebral ischemia did not differ from control animals and this finding was similar with a previous study [50]. In contrast, ischemic rats treated with GTex or EGCG once daily for 7 days lowered SOD activity in comparison with cerebral ischemic rats without treatment. Most studies focused on the changes of oxidation markers 24 h after ischemia [8, 51, 52]. The present study determined oxidation marker activities 7 days after cerebral ischemia and found that GTex and EGCG showed significant inhibition. The protective effects of GTex and EGCG are largely due to their inhibition of some enzymes and antioxidative activities by scavenging free radicals. However, EGCG could be converted to an anthocyaninlike compound followed by cleavage of the gallate moiety by oxidation. Active oxygen including superoxide (O_2_^−^) was produced by EGCG, which could decrease SOD activity by peroxyl radicals formation of superoxide during the inhibitory action [53]. That could be explaining the cause decreased SOD activity in administration of GTex and EGCG once daily for 7 days in the present study. 

GSH is an endogenous antioxidant protecting cells against damage produced by oxygen free radicals. There was a significant decrease in GSH levels 7 days after cerebral ischemia as compared with GSH levels in the sham-operated rats. Treatment with GTex and EGCG once daily for 7 days increased GSH levels in ischemic rats, which may be indicative of neuroprotection. The lower dose of GTex 30 mg/kg was ineffective as there was an insignificant difference between the GTex-treated and I/R (control) rats on the MDA and GSH levels. This result was well correlated with the smaller infarction volume and better functional recovery for higher dose (100, 300 mg/kg) GTex-treated rats than for lower dose (30 mg/kg) GTex-treated or I/R (control) rats.

PTX has been used to treat vascular dementia and multiinfarct dementia in clinical medicine [54, 55] and also proved to slow the progression of dementia [56]. In the present study, PTX was used as a positive control and ameliorated the spatial performance impairment, but did not ameliorate reference memory deficit in cerebral ischemia rats. PTX found to be no antioxidant and anti-lipid peroxidation effects in this study, which is consistent with a previous study [57]. 

Brain inflammation occurs following ischemia-reperfusion [58]. Previous studies showed that activation of BV-2 cells during LPS stimulation could be used to survey the neuroinflammatory effects [59]. Excessive NO and ROS production in the brain contribute to neuronal cell injury processes [60]. Recent studies showed that inhibiting LPS-induced NO production may be neuroprotective [61]. Microglia activation by LPS releases proinflammatory factors, tumor necrosis factor-alpha (TNF-*α*), interleukin 1-beta (IL-1*β*), NO, and superoxide, thus leading to neuronal injury and death [62]. We investigated NO production and iNOS protein expression in BV2 cells treated with EGCG and found that EGCG inhibited LPS-induced NO production and iNOS protein expression in BV-2 cells. Li et al. [30] showed that EGCG inhibited NO production and iNOS protein expression in primary microglia induced by LPS. EGCG could potently inhibit NO and TNF-*α* generation in microglia. Many inflammatory diseases are associated with increased levels of COX-2, another inflammatory factor [59]. In the present study, EGCG inhibited COX-2 protein expression in BV-2 cells. Activated microglia are the primary donor of free radicals and inflammatory factors. 

In summary, GTex and EGCG reduced cerebral infarction and improved learning and memory deficits induced by cerebral ischemia. These effects may involve a reduction in oxidative stress and neuroinflammation induced by ischemia. GTex and EGCG may be efficacious in treating ischemia-induced learning and memory deficits. 

## Figures and Tables

**Table 1:** Composition of GTex.

Component	*μ*g/mg	% of GTex
Total polyphenols	101.80 ± 4.55	10.18%
Polyphenols		
(−)-Epigallocatechin gallate	32.10 ± 0.44	3.21%
(−)-Epigallocatechin	45.96 ± 3.01	4.59%
(−)-Epicatechin gallate	10.62 ± 0.57	1.06%
(−)-Epicatechin	13.11 ± 1.08	1.31%
Caffeine	44.60 ± 0.29	4.46%

**Table 2:** Effect of GTex (p.o.) and EGCG (p.o.) on MDA levels in cortex and hippocampus of ischemia/reperfusion (I/R) rats.

	MDA levels (nmole/mg Protein)	
	Cortex	Hippocampus
Sham	0.73 ± 0.04	0.31 ± 0.03
I/R	1.60 ± 0.19^∗∗∗^	1.03 ± 0.07^∗∗∗^
GTex (30 mg/kg)	1.23 ± 0.19	0.89 ± 0.09
GTex (100 mg/kg)	1.16 ± 0.13	0.54 ± 0.06^##^
GTex (300 mg/kg)	0.65 ± 0.08^###^	0.30 ± 0.03^###^
EGCG (10 mg/kg)	0.96 ± 0.09^##^	0.56 ± 0.11^#^
PTX (100 mg/kg)	1.24 ± 0.06	1.02 ± 0.06

***P < 0.001 compared to the sham group, ^#^ *P* < 0.05, ^##^ *P* < 0.01, ^###^ *P* < 0.001 compared to I/R group (*N* = 6).

**Table 3:** Effect of GTex (p.o.) and EGCG (p.o.) on SOD activities in cortex and hippocampus of ischemia/reperfusion (I/R) rats.

	SOD activity (U/mg Protein)	
	Cortex	Hippocampus
Sham	1.05 ± 0.08	2.06 ± 0.12
I/R	1.08 ± 0.06	2.02 ± 0.08
GTex (30 mg/kg)	1.02 ± 0.15	2.04 ± 0.15
GTex (100 mg/kg)	0.89 ± 0.10	1.90 ± 0.14
GTex (300 mg/kg)	0.69 ± 0.09^#^	1.48 ± 0.10^##^
EGCG (10 mg/kg)	0.72 ± 0.03^#^	1.47 ± 0.08^##^
PTX (100 mg/kg)	1.04 ± 0.1	2.19 ± 0.06

^#^ *P* < 0.05, ^##^ *P* < 0.01 compared to I/R group (*N* = 6).

**Table 4:** Effect of GTex (p.o.) and EGCG (p.o.) on GSH levels in cortex and hippocampus of ischemia/reperfusion (I/R) rats.

	GSH levels (pmole/mg Protein)	
	Cortex	Hippocampus
Sham	20.29 ± 1.12	103.72 ± 5.73
I/R	10.61 ± 1.22^∗∗∗^	63.71 ± 4.83^∗∗∗^
GTex (30 mg/kg)	13.78 ± 1.09	66.88 ± 7.17
GTex (100 mg/kg)	17.80 ± 1.92^##^	96.43 ± 6.74^###^
GTex (300 mg/kg)	18.95 ± 0.88^###^	104.73 ± 7.83^###^
EGCG (10 mg/kg)	17.80 ± 0.98^##^	139.01 ± 8.26^###^
PTX (100 mg/kg)	14.06 ± 1.43	76.09 ± 6.25

***P < 0.001 compared to the sham group, ^##^ *P* < 0.01, ^###^ *P* < 0.001 compared to I/R group (*N* = 6).
